# Ethyl 1-*sec*-butyl-2-(4-fluoro­phen­yl)-1*H*-benzimidazole-5-carboxyl­ate

**DOI:** 10.1107/S1600536811041663

**Published:** 2011-10-12

**Authors:** Natarajan Arumugam, Nurziana Ngah, Shafida Abd Hamid, Aisyah Saad Abdul Rahim

**Affiliations:** a School of Pharmaceutical Sciences, Universiti Sains Malaysia, 11800 USM, Penang, Malaysia; b Kulliyyah of Science, International Islamic University Malaysia, Kuantan Campus, Jalan Istana, Bandar Indera Mahkota, 25200 Kuantan, Pahang, Malaysia

## Abstract

In the title compound, C_20_H_21_FN_2_O_2_, the benzene ring and the benzimidazole ring system are inclined at a dihedral angle of 44.40 (9)°. In the crystal, mol­ecules are linked by inter­molecular C—H⋯O hydrogen bonds, forming a zigzag chain along the *b*-axis direction. An intra­molecular C—H⋯π inter­action is also observed.

## Related literature

For the synthesis of the title compound and related structures, see: Arumugam, Abd Hamid *et al.* (2010 ▶); Arumugam, Abdul Rahim, Osman, Hemamalini & Fun (2010 ▶); Arumugam, Abdul Rahim, Osman, Quah & Fun (2010 ▶). For applications of benzimidazole derivatives, see: Spasov *et al.* (1999 ▶); Easmon *et al.* (2001 ▶); Özden *et al.* (2004 ▶). For bond-length data, see: Allen *et al.* (1987 ▶).
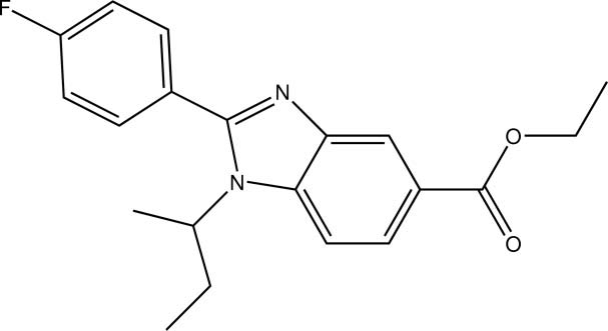


## Experimental

### 

#### Crystal data

C_20_H_21_FN_2_O_2_
*M* *_r_* = 340.39
Monoclinic, 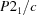

*a* = 10.2249 (16) Å
*b* = 12.3767 (18) Å
*c* = 14.149 (2) Å
β = 93.473 (2)°
*V* = 1787.3 (5) Å^3^
*Z* = 4
Mo *K*α radiation
μ = 0.09 mm^−1^
*T* = 293 K
0.37 × 0.20 × 0.11 mm

#### Data collection

Bruker APEXII DUO CCD diffractometer
Absorption correction: multi-scan (*SADABS*; Bruker, 2009 ▶) *T*_min_ = 0.968, *T*_max_ = 0.990
10465 measured reflections
3130 independent reflections
2342 reflections with *I* > 2σ(*I*)
*R*_int_ = 0.027

#### Refinement

*R*[*F* ^2^ > 2σ(*F* ^2^)] = 0.047
*wR*(*F* ^2^) = 0.150
*S* = 1.05
3130 reflections
229 parameters
H-atom parameters constrained
Δρ_max_ = 0.21 e Å^−3^
Δρ_min_ = −0.20 e Å^−3^

### 

Data collection: *APEX2* (Bruker, 2009 ▶); cell refinement: *SAINT* (Bruker, 2009 ▶); data reduction: *SAINT*; program(s) used to solve structure: *SHELXTL* (Sheldrick, 2008 ▶); program(s) used to refine structure: *SHELXTL*; molecular graphics: *SHELXTL*; software used to prepare material for publication: *SHELXTL* and *PLATON* (Spek, 2009 ▶).

## Supplementary Material

Crystal structure: contains datablock(s) global, I. DOI: 10.1107/S1600536811041663/is2787sup1.cif

Structure factors: contains datablock(s) I. DOI: 10.1107/S1600536811041663/is2787Isup2.hkl

Supplementary material file. DOI: 10.1107/S1600536811041663/is2787Isup3.cml

Additional supplementary materials:  crystallographic information; 3D view; checkCIF report

## Figures and Tables

**Table 1:** Hydrogen-bond geometry (Å, °) *Cg*1 is the centroid of the N1/C7/N2/C1/C6 ring.

*D*—H⋯*A*	*D*—H	H⋯*A*	*D*⋯*A*	*D*—H⋯*A*
C5—H5⋯O1^i^	0.93	2.53	3.452 (3)	169
C20—H20*C*⋯O1^i^	0.96	2.59	3.485 (4)	154
C19—H19*A*⋯*Cg*1	0.96	2.82	3.400 (3)	121

Symmetry code: (i) 
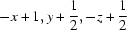
.

**Fig. 1.**
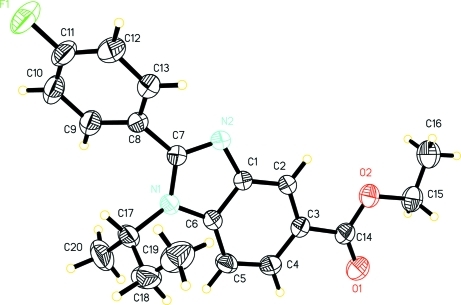
The molecular structure of the title compound, with the atom-numbering scheme and 50% probability displacement ellipsoids for non-H atoms.

**Fig. 2.**
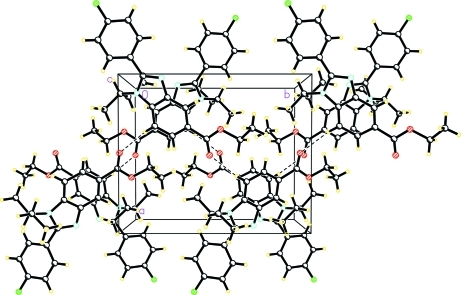
A molecular packing diagram of the title compound, viewed down the c axis.

## supplementary crystallographic information

### Comment

The synthesis of benzimidazole heterocycles is ever fascinating since they promise a wide spectrum of pharmacological activities such as antibacterial (Özden *et al.*, 2004), anticancer (Easmon *et al.*, 2001) and antifungal (Spasov *et al.*, 1999). As the benzimidazole derivative is of much importance, we have undertaken the X-ray crystal structure determination of the title compound.

The title compound (Fig. 1) is similar to those previously reported ethyl-1-*sec*-butyl-2-(4-chlorophenyl)-1*H*-benzimidazole-5-carboxylate (Arumugam, Abdul Rahim, Osman, Quah & Fun, 2010) except the fluorine atom is attached at the *para* position of the phenyl ring. The phenyl (C8/C9/C10/ C11/C12/C13) and benzimidazole (N1/N2/C1/C2/C3/C4/C5) fragments are essentially planar with maximum deviation is 0.005 (2)Å for atom C2. Both fragments are inclined to each other by 44.40 (9)°. The bond lengths are in normal ranges (Allen *et al.*, 1987) and in agreement to those reported by Arumugam *et al.* (Arumugam, Abd Hamid *et al.*, 2010; Arumugam, Abdul Rahim, Osman, Hemamalini & Fun, 2010; Arumugam, Abdul Rahim, Osman, Quah & Fun, 2010). In the crystal structure (Fig. 2), the molecules are linked by intermolecular C5—H5···O1^i^ and C20—H20C···O1^i^ hydrogen bonds (symmetry codes as in Table 1) to form a zigzag chain along the *b* axis. The molecular structure is further stabilized by an intramolecular C—H···*Cg*1 (Table 1) interaction; *Cg*1 is the centroid of the N1/C7/N2/C1/C6 ring.

### Experimental

A solution of ethyl-3-amino-4-(*sec*-butylamino) benzoate (1.0 mmol) and sodium bisulfite adduct of 4-fluorobenzaldehyde (3.5 mmol) in DMF was treated under microwave conditions at 130 °C for 2 minutes. The reaction mixture was diluted in EtOAc (20 ml) and washed with H_2_O (20 ml). The organic layer was collected and dried with Na_2_SO_4_. The solvent was evaporated in *vacuo* to afford a crude product. Recrystallization of the crude product gave the title compound as colourless crystal.

### Refinement

All H atoms were positioned geometrically and refined using a riding model, with C—H = 0.93–0.98 Å, and with *U*_iso_(H)= 1.2 or 1.5*U*_eq_(C). The rotating group model was applied for methyl groups.

### Crystal data

**Table:**

C_20_H_21_FN_2_O_2_	*F*(000) = 720
*M**_r_* = 340.39	*D*_x_ = 1.265 Mg m^−^^3^
Monoclinic, *P*2_1_/*c*	Mo *K*α radiation, λ = 0.71073 Å
Hall symbol: -P 2ybc	Cell parameters from 2853 reflections
*a* = 10.2249 (16) Å	θ = 2.0–25.0°
*b* = 12.3767 (18) Å	µ = 0.09 mm^−^^1^
*c* = 14.149 (2) Å	*T* = 293 K
β = 93.473 (2)°	Block, colourless
*V* = 1787.3 (5) Å^3^	0.37 × 0.20 × 0.11 mm
*Z* = 4	

### Data collection

**Table:**

Bruker APEXII DUO CCD diffractometer	3130 independent reflections
Radiation source: fine-focus sealed tube	2342 reflections with *I* > 2σ(*I*)
graphite	*R*_int_ = 0.027
Detector resolution: 83.66 pixels mm^-1^	θ_max_ = 25.0°, θ_min_ = 2.0°
φ and ω scans	*h* = −12→12
Absorption correction: multi-scan (*SADABS*; Bruker, 2009)	*k* = −14→14
*T*_min_ = 0.968, *T*_max_ = 0.990	*l* = −16→16
10465 measured reflections	

### Refinement

**Table:**

Refinement on *F*^2^	Primary atom site location: structure-invariant direct methods
Least-squares matrix: full	Secondary atom site location: difference Fourier map
*R*[*F*^2^ > 2σ(*F*^2^)] = 0.047	Hydrogen site location: inferred from neighbouring sites
*wR*(*F*^2^) = 0.150	H-atom parameters constrained
*S* = 1.05	*w* = 1/[σ^2^(*F*_o_^2^) + (0.0825*P*)^2^ + 0.313*P*] where *P* = (*F*_o_^2^ + 2*F*_c_^2^)/3
3130 reflections	(Δ/σ)_max_ = 0.001
229 parameters	Δρ_max_ = 0.21 e Å^−^^3^
0 restraints	Δρ_min_ = −0.20 e Å^−^^3^

### Special details

**Table:**

Geometry. All e.s.d.'s (except the e.s.d. in the dihedral angle between two l.s. planes) are estimated using the full covariance matrix. The cell e.s.d.'s are taken into account individually in the estimation of e.s.d.'s in distances, angles and torsion angles; correlations between e.s.d.'s in cell parameters are only used when they are defined by crystal symmetry. An approximate (isotropic) treatment of cell e.s.d.'s is used for estimating e.s.d.'s involving l.s. planes.
Refinement. Refinement of *F*^2^ against ALL reflections. The weighted *R*-factor *wR* and goodness of fit *S* are based on *F*^2^, conventional *R*-factors *R* are based on *F*, with *F* set to zero for negative *F*^2^. The threshold expression of *F*^2^ > σ(*F*^2^) is used only for calculating *R*-factors(gt) *etc*. and is not relevant to the choice of reflections for refinement. *R*-factors based on *F*^2^ are statistically about twice as large as those based on *F*, and *R*- factors based on ALL data will be even larger.

### Fractional atomic coordinates and isotropic or equivalent isotropic displacement parameters (Å^2^)

**Table:**

	*x*	*y*	*z*	*U*_iso_*/*U*_eq_	
F1	1.45030 (16)	1.11874 (14)	0.07109 (13)	0.1053 (6)	
O1	0.49279 (17)	0.52863 (13)	0.17567 (13)	0.0764 (5)	
O2	0.65827 (16)	0.47066 (12)	0.09413 (12)	0.0696 (5)	
N1	0.89771 (15)	0.92002 (12)	0.18887 (11)	0.0456 (4)	
N2	0.98670 (15)	0.78337 (12)	0.10912 (11)	0.0468 (4)	
C1	0.86373 (18)	0.75303 (14)	0.13674 (12)	0.0421 (4)	
C2	0.79676 (19)	0.65643 (15)	0.12078 (13)	0.0458 (5)	
H2	0.8334	0.6006	0.0872	0.055*	
C3	0.67392 (19)	0.64541 (15)	0.15618 (13)	0.0467 (5)	
C4	0.6195 (2)	0.73015 (17)	0.20597 (15)	0.0537 (5)	
H4	0.5372	0.7208	0.2293	0.064*	
C5	0.68326 (19)	0.82662 (17)	0.22166 (15)	0.0542 (5)	
H5	0.6456	0.8827	0.2543	0.065*	
C6	0.80724 (18)	0.83677 (15)	0.18631 (13)	0.0439 (5)	
C7	1.00272 (18)	0.88255 (15)	0.14109 (12)	0.0428 (4)	
C8	1.11991 (18)	0.94762 (15)	0.12502 (13)	0.0443 (5)	
C9	1.1114 (2)	1.05455 (17)	0.09460 (14)	0.0557 (5)	
H9	1.0297	1.0875	0.0865	0.067*	
C10	1.2223 (3)	1.11195 (19)	0.07637 (16)	0.0658 (6)	
H10	1.2166	1.1834	0.0561	0.079*	
C11	1.3408 (2)	1.0617 (2)	0.08865 (17)	0.0672 (6)	
C12	1.3540 (2)	0.9576 (2)	0.11717 (18)	0.0697 (7)	
H12	1.4361	0.9253	0.1240	0.084*	
C13	1.2422 (2)	0.90061 (18)	0.13581 (16)	0.0582 (6)	
H13	1.2496	0.8293	0.1560	0.070*	
C14	0.5983 (2)	0.54440 (17)	0.14437 (14)	0.0528 (5)	
C15	0.5918 (3)	0.36793 (19)	0.0794 (2)	0.0798 (8)	
H15A	0.5018	0.3795	0.0559	0.096*	
H15B	0.5915	0.3282	0.1385	0.096*	
C16	0.6626 (3)	0.3076 (2)	0.0104 (2)	0.0967 (10)	
H16A	0.6615	0.3474	−0.0479	0.145*	
H16B	0.6213	0.2387	−0.0007	0.145*	
H16C	0.7515	0.2970	0.0342	0.145*	
C17	0.8973 (2)	1.01576 (17)	0.25156 (16)	0.0608 (6)	
H17	0.9825	1.0513	0.2477	0.073*	
C18	0.8868 (3)	0.9822 (2)	0.35314 (18)	0.0803 (8)	
H18A	0.8993	1.0451	0.3935	0.096*	
H18B	0.7994	0.9545	0.3610	0.096*	
C19	0.9833 (3)	0.8994 (3)	0.38342 (18)	0.0906 (9)	
H19A	0.9644	0.8337	0.3492	0.136*	
H19B	0.9789	0.8864	0.4500	0.136*	
H19C	1.0696	0.9240	0.3708	0.136*	
C20	0.7917 (3)	1.0977 (2)	0.2147 (3)	0.0974 (10)	
H20A	0.7997	1.1099	0.1483	0.146*	
H20B	0.8036	1.1647	0.2483	0.146*	
H20C	0.7062	1.0693	0.2246	0.146*	

### Atomic displacement parameters (Å^2^)

**Table:**

	*U*^11^	*U*^22^	*U*^33^	*U*^12^	*U*^13^	*U*^23^
F1	0.0832 (11)	0.1053 (12)	0.1309 (14)	−0.0492 (9)	0.0352 (10)	0.0009 (10)
O1	0.0610 (10)	0.0656 (11)	0.1054 (13)	−0.0198 (8)	0.0281 (9)	−0.0043 (9)
O2	0.0676 (10)	0.0470 (9)	0.0966 (11)	−0.0210 (7)	0.0245 (9)	−0.0150 (8)
N1	0.0434 (9)	0.0390 (9)	0.0552 (9)	−0.0022 (7)	0.0100 (7)	−0.0086 (7)
N2	0.0453 (9)	0.0410 (9)	0.0558 (9)	−0.0032 (7)	0.0156 (7)	−0.0043 (7)
C1	0.0423 (10)	0.0366 (10)	0.0481 (10)	−0.0005 (8)	0.0095 (8)	−0.0007 (8)
C2	0.0474 (10)	0.0364 (10)	0.0545 (11)	0.0001 (8)	0.0126 (9)	−0.0029 (8)
C3	0.0445 (10)	0.0431 (11)	0.0530 (11)	−0.0042 (8)	0.0072 (8)	0.0024 (8)
C4	0.0403 (10)	0.0556 (12)	0.0666 (12)	−0.0026 (9)	0.0145 (9)	−0.0033 (10)
C5	0.0450 (11)	0.0503 (12)	0.0686 (13)	0.0014 (9)	0.0146 (9)	−0.0131 (10)
C6	0.0419 (10)	0.0385 (10)	0.0519 (10)	0.0004 (8)	0.0072 (8)	−0.0039 (8)
C7	0.0449 (10)	0.0394 (10)	0.0448 (10)	−0.0021 (8)	0.0074 (8)	−0.0016 (8)
C8	0.0459 (10)	0.0433 (11)	0.0446 (9)	−0.0058 (8)	0.0095 (8)	−0.0043 (8)
C9	0.0587 (13)	0.0506 (12)	0.0579 (12)	−0.0065 (10)	0.0043 (10)	0.0044 (9)
C10	0.0820 (17)	0.0535 (13)	0.0625 (13)	−0.0223 (12)	0.0088 (12)	0.0089 (10)
C11	0.0623 (14)	0.0713 (16)	0.0702 (14)	−0.0285 (12)	0.0205 (11)	−0.0040 (12)
C12	0.0474 (13)	0.0732 (16)	0.0897 (17)	−0.0075 (11)	0.0139 (11)	−0.0017 (13)
C13	0.0524 (12)	0.0505 (12)	0.0731 (14)	−0.0045 (10)	0.0143 (10)	0.0022 (10)
C14	0.0500 (12)	0.0478 (12)	0.0613 (12)	−0.0066 (9)	0.0099 (10)	0.0027 (9)
C15	0.0873 (18)	0.0516 (14)	0.1029 (19)	−0.0304 (13)	0.0256 (15)	−0.0133 (13)
C16	0.109 (2)	0.0612 (16)	0.123 (2)	−0.0270 (16)	0.0293 (19)	−0.0215 (16)
C17	0.0572 (13)	0.0468 (12)	0.0804 (15)	−0.0078 (10)	0.0197 (11)	−0.0239 (11)
C18	0.0765 (18)	0.095 (2)	0.0720 (16)	−0.0237 (15)	0.0244 (13)	−0.0351 (14)
C19	0.098 (2)	0.114 (2)	0.0592 (15)	−0.0281 (19)	0.0050 (14)	0.0001 (15)
C20	0.0765 (18)	0.0528 (15)	0.165 (3)	0.0089 (13)	0.0213 (18)	−0.0266 (16)

### Geometric parameters (Å, °)

**Table:**

F1—C11	1.359 (2)	C10—C11	1.363 (4)
O1—C14	1.207 (2)	C10—H10	0.9300
O2—C14	1.329 (3)	C11—C12	1.355 (4)
O2—C15	1.451 (3)	C12—C13	1.382 (3)
N1—C7	1.383 (2)	C12—H12	0.9300
N1—C6	1.384 (2)	C13—H13	0.9300
N1—C17	1.480 (2)	C15—C16	1.456 (4)
N2—C7	1.315 (2)	C15—H15A	0.9700
N2—C1	1.391 (2)	C15—H15B	0.9700
C1—C2	1.390 (3)	C16—H16A	0.9600
C1—C6	1.396 (2)	C16—H16B	0.9600
C2—C3	1.387 (3)	C16—H16C	0.9600
C2—H2	0.9300	C17—C18	1.506 (4)
C3—C4	1.397 (3)	C17—C20	1.549 (4)
C3—C14	1.474 (3)	C17—H17	0.9800
C4—C5	1.372 (3)	C18—C19	1.468 (4)
C4—H4	0.9300	C18—H18A	0.9700
C5—C6	1.396 (3)	C18—H18B	0.9700
C5—H5	0.9300	C19—H19A	0.9600
C7—C8	1.473 (3)	C19—H19B	0.9600
C8—C13	1.379 (3)	C19—H19C	0.9600
C8—C9	1.393 (3)	C20—H20A	0.9600
C9—C10	1.376 (3)	C20—H20B	0.9600
C9—H9	0.9300	C20—H20C	0.9600
			
C14—O2—C15	116.83 (18)	C8—C13—H13	119.4
C7—N1—C6	105.97 (14)	C12—C13—H13	119.4
C7—N1—C17	126.27 (16)	O1—C14—O2	122.46 (19)
C6—N1—C17	125.85 (16)	O1—C14—C3	124.7 (2)
C7—N2—C1	104.55 (15)	O2—C14—C3	112.79 (17)
C2—C1—N2	129.17 (16)	O2—C15—C16	107.4 (2)
C2—C1—C6	120.37 (17)	O2—C15—H15A	110.2
N2—C1—C6	110.45 (15)	C16—C15—H15A	110.2
C3—C2—C1	118.30 (17)	O2—C15—H15B	110.2
C3—C2—H2	120.8	C16—C15—H15B	110.2
C1—C2—H2	120.9	H15A—C15—H15B	108.5
C2—C3—C4	120.33 (18)	C15—C16—H16A	109.5
C2—C3—C14	121.51 (18)	C15—C16—H16B	109.5
C4—C3—C14	118.15 (18)	H16A—C16—H16B	109.5
C5—C4—C3	122.36 (19)	C15—C16—H16C	109.5
C5—C4—H4	118.8	H16A—C16—H16C	109.5
C3—C4—H4	118.8	H16B—C16—H16C	109.5
C4—C5—C6	116.93 (18)	N1—C17—C18	110.73 (18)
C4—C5—H5	121.5	N1—C17—C20	110.4 (2)
C6—C5—H5	121.5	C18—C17—C20	114.4 (2)
N1—C6—C1	105.64 (16)	N1—C17—H17	106.9
N1—C6—C5	132.65 (17)	C18—C17—H17	106.9
C1—C6—C5	121.70 (17)	C20—C17—H17	106.9
N2—C7—N1	113.38 (16)	C19—C18—C17	112.7 (2)
N2—C7—C8	122.88 (16)	C19—C18—H18A	109.1
N1—C7—C8	123.72 (16)	C17—C18—H18A	109.1
C13—C8—C9	118.32 (18)	C19—C18—H18B	109.1
C13—C8—C7	119.53 (18)	C17—C18—H18B	109.1
C9—C8—C7	122.07 (18)	H18A—C18—H18B	107.8
C10—C9—C8	120.8 (2)	C18—C19—H19A	109.5
C10—C9—H9	119.6	C18—C19—H19B	109.5
C8—C9—H9	119.6	H19A—C19—H19B	109.5
C11—C10—C9	118.5 (2)	C18—C19—H19C	109.5
C11—C10—H10	120.8	H19A—C19—H19C	109.5
C9—C10—H10	120.8	H19B—C19—H19C	109.5
C12—C11—F1	118.7 (2)	C17—C20—H20A	109.5
C12—C11—C10	122.9 (2)	C17—C20—H20B	109.5
F1—C11—C10	118.4 (2)	H20A—C20—H20B	109.5
C11—C12—C13	118.3 (2)	C17—C20—H20C	109.5
C11—C12—H12	120.9	H20A—C20—H20C	109.5
C13—C12—H12	120.9	H20B—C20—H20C	109.5
C8—C13—C12	121.2 (2)		
			
C7—N2—C1—C2	179.31 (19)	N1—C7—C8—C13	138.2 (2)
C7—N2—C1—C6	−0.2 (2)	N2—C7—C8—C9	133.3 (2)
N2—C1—C2—C3	179.81 (18)	N1—C7—C8—C9	−45.0 (3)
C6—C1—C2—C3	−0.7 (3)	C13—C8—C9—C10	−0.4 (3)
C1—C2—C3—C4	0.4 (3)	C7—C8—C9—C10	−177.26 (18)
C1—C2—C3—C14	−178.35 (17)	C8—C9—C10—C11	0.1 (3)
C2—C3—C4—C5	0.3 (3)	C9—C10—C11—C12	0.6 (4)
C14—C3—C4—C5	179.11 (19)	C9—C10—C11—F1	−179.65 (19)
C3—C4—C5—C6	−0.7 (3)	F1—C11—C12—C13	179.3 (2)
C7—N1—C6—C1	0.10 (19)	C10—C11—C12—C13	−0.9 (4)
C17—N1—C6—C1	−164.91 (18)	C9—C8—C13—C12	0.1 (3)
C7—N1—C6—C5	−179.7 (2)	C7—C8—C13—C12	176.99 (19)
C17—N1—C6—C5	15.3 (3)	C11—C12—C13—C8	0.6 (3)
C2—C1—C6—N1	−179.50 (16)	C15—O2—C14—O1	−1.0 (3)
N2—C1—C6—N1	0.1 (2)	C15—O2—C14—C3	179.5 (2)
C2—C1—C6—C5	0.3 (3)	C2—C3—C14—O1	177.9 (2)
N2—C1—C6—C5	179.88 (18)	C4—C3—C14—O1	−1.0 (3)
C4—C5—C6—N1	−179.9 (2)	C2—C3—C14—O2	−2.6 (3)
C4—C5—C6—C1	0.4 (3)	C4—C3—C14—O2	178.55 (18)
C1—N2—C7—N1	0.3 (2)	C14—O2—C15—C16	170.5 (2)
C1—N2—C7—C8	−178.22 (16)	C7—N1—C17—C18	−110.7 (2)
C6—N1—C7—N2	−0.2 (2)	C6—N1—C17—C18	51.3 (3)
C17—N1—C7—N2	164.68 (18)	C7—N1—C17—C20	121.5 (2)
C6—N1—C7—C8	178.24 (17)	C6—N1—C17—C20	−76.5 (3)
C17—N1—C7—C8	−16.8 (3)	N1—C17—C18—C19	50.7 (3)
N2—C7—C8—C13	−43.5 (3)	C20—C17—C18—C19	176.3 (2)

### Hydrogen-bond geometry (Å, °)

**Table:**

*Cg*1 is the centroid of the N1/C7/N2/C1/C6 ring.

**Table:**

*D*—H···*A*	*D*—H	H···*A*	*D*···*A*	*D*—H···*A*
C5—H5···O1^i^	0.93	2.53	3.452 (3)	169
C20—H20C···O1^i^	0.96	2.59	3.485 (4)	154
C19—H19A···Cg1	0.96	2.82	3.400 (3)	121

Symmetry codes: (i) −*x*+1, *y*+1/2, −*z*+1/2.
